# Telomeric and sub-telomeric regions undergo rapid turnover within a *Streptomyces* population

**DOI:** 10.1038/s41598-020-63912-w

**Published:** 2020-05-07

**Authors:** Abdoul-Razak Tidjani, Cyril Bontemps, Pierre Leblond

**Affiliations:** Université de Lorraine, INRAE, DynAMic, F-54000 Nancy, France

**Keywords:** Bacterial genomics, Microbial ecology, Bacterial genetics, Evolutionary genetics, Molecular evolution, Genome, Genomic instability, Genomics

## Abstract

Genome dynamics was investigated within natural populations of the soil bacterium *Streptomyces*. The exploration of a set of closely related strains isolated from micro-habitats of a forest soil exhibited a strong diversity of the terminal structures of the linear chromosome, *i.e*. terminal inverted repeats (TIRs). Large insertions, deletions and translocations could be observed along with evidence of transfer events between strains. In addition, the telomere and its cognate terminal protein complexes required for terminal replication and chromosome maintenance, were shown to be variable within the population probably reflecting telomere exchanges between the chromosome and other linear replicons (*i.e*., plasmids). Considering the close genetic relatedness of the strains, these data suggest that the terminal regions are prone to a high turnover due to a high recombination associated with extensive horizontal gene transfer.

## Introduction

*Streptomyces* are filamentous soil bacteria producing a large array of extracellular enzymes and metabolites involved in biogeochemical cycles (*i.e*. degradation of organic matter, mineral weathering etc^1,2^; or in mediating complex biotic interactions with the wider soil organism community. The biochemical arsenal of *Streptomyces* has been extensively exploited for industry^3,4^ and medical applications. The latter including the production of antibiotics, herbicides, or antitumor compounds.

*Streptomyces* possess genomes amongst the largest reported for bacteria with a chromosome range of 6 Mb to 12 Mb. Along with *Borrelia burgdorferi*^5^, *Agrobacterium tumefaciens*^6^ and other Actinobacteria such as *Rhodoccocus*^7^ they share the rare characteristic of chromosome linearity. The linear chromosomal DNA is complemented by the presence of linear and circular plasmids. *Streptomyces* linear replicons share the same invertron structure, *i.e*., the presence of terminal redundancies of variable sizes (TIRs for Terminal Inverted Repeats). TIRs are variable in size, being as short as several tens of nucleotides and serve as telomeres (see below) such as in *S. avermitilis*^8^, or they can reach up to 1 Mb such as in *S. coelicolor* A3(2)^9^ or larger (1.4 Mb in *S. ambofaciens*^10^. Variability of the TIR size was reported at the interspecific^11^ and intraspecific levels^9,10,12,13^, however, to date, no study has investigated the TIR variability over short evolutionary scales or at the population level.

Chromosomal replication of linear bacterial chromosomes is initiated at *oriC* replicating simultaneously towards both ends of the chromosome, duplicating the DNA except at the extreme ends of the parental 3′ DNA strands, due to a failure to prime the discontinuous replication process of the lagging strand^14^. This classical end-of-replication problem of linear chromosomes is overcome thanks to a complex of terminal proteins called TP-TAP that binds telomeres. The TP/TAP complex, through TAP, allows priming DNA replication from the 3′ end and filling of the replication gap. At the end of the ‘end-patching’ the TP protein remains covalently attached to the 5′ end of the DNA strand ensuring the protection against nucleases and recruits TAP for the next replication round^15^.

The bacterial telomeres constitute a functional analogue of eukaryotic telomeres by preventing chromosomal ends from progressive shortening and loss of genetic information through successive replication rounds. In *Streptomyces*, they consist of a *cis*-acting sequence of about 160 nucleotides at the very end of the chromosome that form critical stem-loops involved in terminal structures known as ‘rabbit ears’ or ‘clover leaves’ models. The telomere sequences of the chromosomes of the models *Streptomyces coelicolor* A3(2) and *S. avermitilis* were defined as ‘archetypal’^16–18^. They are typified by a conserved 13 palindromic motif at the very end of the chromosome (Palindrome I) as well as five more internal but less strictly conserved palindromes. These six palindromes constitute the minimal telomere sequence needed for maintenance of plasmid pSLA2 of *Streptomyces rochei*^19^. The central parts of the palindromes are characterized by a 3-nucleotide typical DNA motif, mostly 5′-GCA-3′. This ‘sheared purine-purine’ pairing confers a resistance to single-strand nucleases^20^ and may play a protective role when these extremities are exposed during replication. ‘Atypical’ telomeres (*e.g*. including those of plasmids such as SCP1 of *S. coelicolor*^21^ and those of the chromosome of *Streptomyces griseus*^17^; were also described and consist of sequences that are heterogeneous in size and sequence in comparison with the archetypal telomeres. In contrast they also exhibit either 4-nucleotide loops (such as those of plasmid SCP1) or 3-nucleotide ones (*e.g*. 5′-GCA-3′ in *S. griseus* 13350). Although variable, these telomeres support terminal replication and probably recruit different terminal proteins (TPs).

Most TP/TAP complexes are encoded as an operon which is often located in a terminal position, i.e. within several hundreds of kilobases of the ends of the chromosome (over a chromosome size of ~8 Mb). The adopted nomenclature indicates that terminal proteins that cap archetypical telomeres are designated as archetypical and non-archetypal terminal proteins cap non-archetypal telomeres. Archetypical TPs include a helix-turn-helix (HTH) domain and a nuclear localization signal (NLS) which has been shown to be efficient to targeting TPs to human and plant nuclei^22^. The non-archetypal TP/TAP systems share some of these features, for instance, the Tac-Tpc of *S. coelicolor* A3(2) also has NLS and a HTH binding domains. However, in comparison with the archetypical TP/TAP systems, they are heterogeneous in size and sequences (for example, GtpA of *S. griseus* exhibits 18% amino acid identity with archetypical Tpg proteins^18^.

Previously we have isolated and fully sequenced the genomes of a eleven conspecific and sympatric strains from a *Streptomyces* population isolated at the micro-scale^23^, demonstrating they share close phylogenetic relationships (identical 16S rRNA gene sequences and weak MLSA divergence) thus forming a population derived from a recent common ancestor. As a consequence of this, the genome divergence within this population is derived from genome rearrangements and gene acquisitions that occurred over a relatively short evolutionary period. Moreover, a gradient of insertion and deletion events was revealed towards the chromosome end supporting the hypothesis that they act as hotspots of recombination and/or better tolerate DNA rearrangements^23^. This strain collection enables to study recent molecular events (recombination) that impact on chromosome structure. Here, given the features of the chromosome extremities (*i.e*. terminal inverted repeats (TIRs) and telomeres) we investigated these strains which provide an ideal system to study chromosome plasticity in an ecological context. We show that the ends of the linear replicons are highly variable, implying a high recombination activity between chromosomal ends as well as acquisition and loss of terminal replication machineries including telomeres and the TAP/TP partner complex.

## Materials and Methods

### Terminal inverted repeats (TIRs) identification

Genome sequencing of the *Streptomyces* chromosomes was previously reported^24^. It consists in one scaffold per chromosome and per extra-chromosomal element when present. The terminal inverted repeats of the chromosomes and linear plasmids were determined *in silico* (CLC Genomics Workbench 6.0, Qiagen) by mapping the Illumina sequencing reads against the reference sequence of the corresponding replicon. The TIRs were determined as the terminal regions with a number of reads twice than the central region of each replicon.

### Telomere identification

The previously published genome sequences (Table 1) include a single copy of the terminal inverted repeats (either in 5′ or in 3′ depending on the assembly). Hence, large duplicated sequences are present only once in the assembly (with a double sequencing depth). After identification of the TIRs, their extremities were extended using the Illumina sequencing read batch using CLC Genomics Workbench (v6.0, Qiagen) in order to find or complete the telomere sequence; for nine of the 11 strains as well as for the three linear plasmids (pRLB1-9.2, pS1D4-14.1, pS1D4-20.1), this procedure allowed to extend the sequence and identify telomere sequences. Updated versions of the chromosomes and plasmid sequence are accessible under the previous accession numbers (24).

**Table 1:**
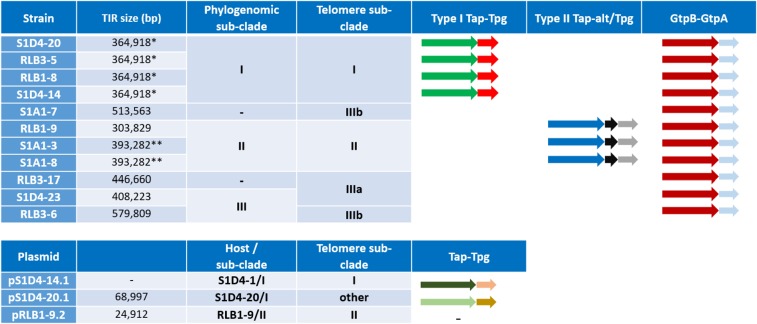
Size of the Terminal Inverted Repeats (TIRs) and distribution of the telomere types and terminal protein encoding genes on the different replicons.

^a^Phylogenomic and telomere sub-clades defined in Fig. 1. The different Tap-Tpg and GtpB-GtpA operons are depicted with arrows. The type I Tap-Tpg and the GtpB-GtpA systems had two open reading frames (ORFs) and the type II Tap-Tpg had three. Within a model, similar colours of the ORFs between strains indicated a sequence identity superior at 98% aa. Differences in colour of plasmid ORFs in type I Tap-Tpg indicated that the sequences are related, but more distant with a sequence identity of 80%. — symbol indicated that no Tap-Tpg system was identified.

*,**: the assessement uncertainty (circa 300 bp, i.e., a read length) did not allow to conclude to a size difference; —: TIR undetected.

### Annotation, DNA fold prediction and phylogenetic analyses

The CDS prediction and functional annotation in the TIR sequences was performed using RAST^25^. The secondary structures of the telomeres were constructed using the *mfold* Web Server^26^ (http://unafold.rna.albany.edu/?q=mfold/DNA-Folding-Form) with a folding temperature of 30 °C in 1 M NaCl. Nuclear Localization Sites (NLS) and Helix-Turn-Helix (HTH) domains were predicted with cNLS mapper server (http://nls-mapper.iab.keio.ac.jp/cgi-bin/NLS_Mapper_y.cgi)^27^ and with the HTH motif prediction software of the Prabi server (https://npsa-prabi.ibcp.fr/) respectively^28^. Conserved domains in Tap-alt were identified with CDsearch (https://www.ncbi.nlm.nih.gov/)^29^. Sequence alignments were performed with ClustalW Multiple alignment algorithm^30^ and the software BioEdit (27). The telomere phylogenetic tree was constructed and edited with MEGA X^31^ using a Neighbour Joining (NJ) method with a K2P correction model. All positions with <80% site coverage were eliminated and support for the tree branches was estimated with 100 bootstrap replicates.

To identify *tap-tpg* and *gtpB-gtpA* homologues, the amino-acids sequences of Tap and Tpg of *Streptomyces coelicolor* A3(2)*, Streptomyces lidycus A02, S. lidycus 103, Streptomyces lividans* (accession number AAL05040.1), *Streptomyces avermitilis* (WP_010988960.1, WP_010988961.1) and those of the GtpB and GtpA of *Streptomyces griseus* (SGR RS00475, SGR_RS00470) were used as a query sequences in Blast^32^. Homologues were identified with a cutoff of 50% of sequence identity and a minimum coverage of 98% between the sequences.

### Comparative genomics and homologous recombination events detection

The comparison of the 11 TIRs was performed with the software Progressive Mauve tool (multiple alignment of conserved genomic sequence with rearrangements^33^; with default parameters. Recombination events were detected with the RDP4 v4.97 program^34^ in a two-step procedure as described in González-Torres *et al*.^35^. First, a full exploratory recombination scan using the nine methods available was performed. The detected events were then rescanned with the RDP, GENECONV, MaxCHI, Chimera and 3Seq algorithms and only events positively detected by three of these five methods were considered.

## Results and Discussion

### Intraspecific variability at the chromosomal ends

The first step of our investigation was the detection and delimitation of the TIRs. *De novo* assemblies performed following next generation sequencing approaches (NGS) do not allow identifying such large intragenomic duplications (*i.e*., duplications larger than the read size). However, the size of TIRs can be deduced from the analysis of read coverage^36^. Hence, a twofold quantity of sequencing reads can be aligned to regions of the assembly that are readily duplicated in the genome. Mapping of the bulk reads onto the genomic sequence obtained at the assembler output is thus an efficient way to precisely detect the limits and extent of the terminal duplications (see Material and Methods section).

The size of the TIRs ranged from 303 kb to 579 kb for chromosomes (Table 1). In addition, among the seven extrachromosomal elements detected in individuals of the population, three (pRLB1–9.2, pS1D4-20.1 and pS1D4-14.1) were linear. The two first possessed TIRs of 24 kb and 68 kb respectively, but no TIR was detected for pS1D4-14.1. Telomeres were found at the extremities of all three replicons (see below).

**Figure 1:**
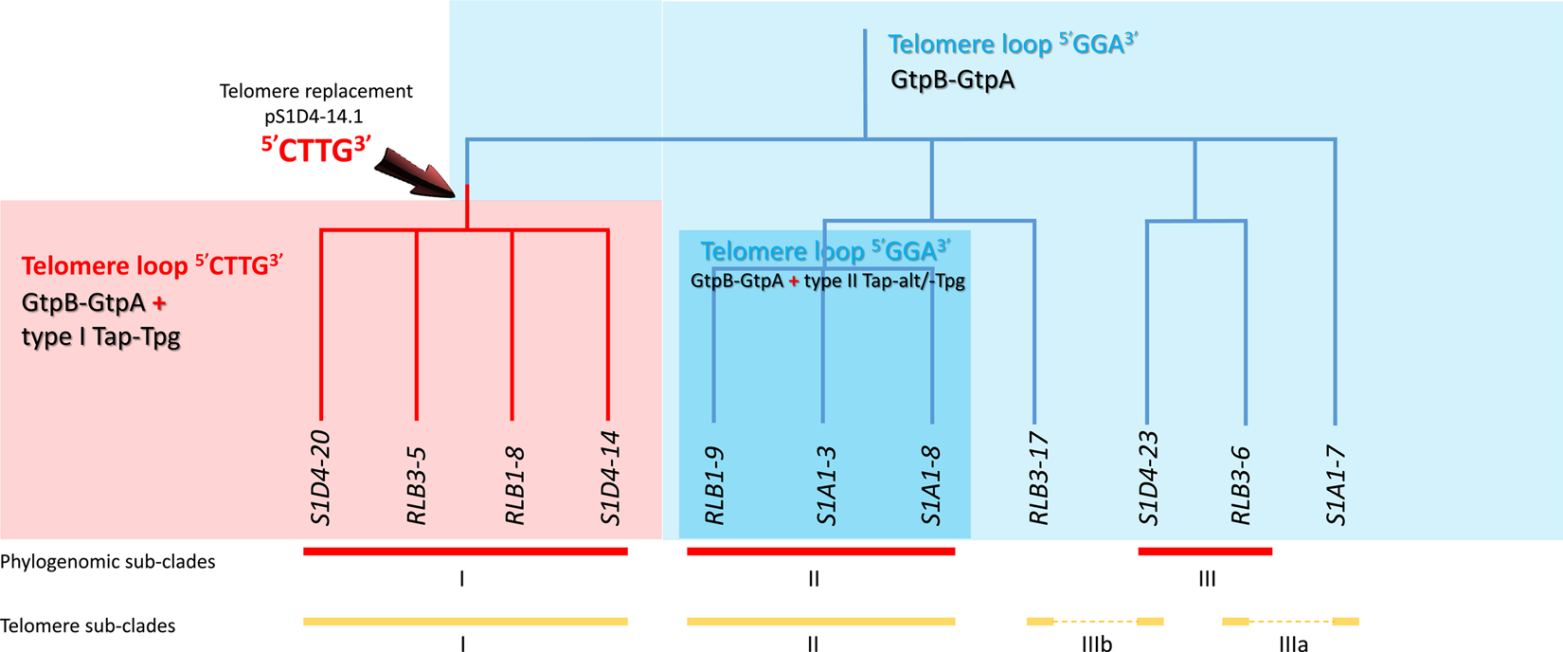
Telomere and terminal protein distribution among the *Streptomyces* population. A maximum likelihood phylogenomic tree of the 11 strains was built based on 5,149,602 nucleotide positions. Heavy lines indicate branches supported by bootstrap values >80% (100 replicates). According to the tree topology and bootstrap values, three phylogenomic sub-clades (I, II and III) were defined. The strains belonging to a same phylogenomic sub-clade are connected with a red bar. The yellow bars connect stains harbouring identical or highly closely related telomere sequences (more than 92% sequence identity). The dashed yellow lines indicate incongruences between the phylogenomic tree and the distribution of similar telomeres. The coloured backgrounds represent the distribution of the chromosomal telomere loops and of the different *tap*-*tpg*/*gtpB*-*gtpA* systems. The light blue background encompasses strains for which only the *gtpB*-*gtpA* system was identified. Their chromosomal telomeres are typified with a ^5′^GGA^3′^ loop. A parsimonious evolutionary scenario suggest that the ancestor of the population shared the same combination. The turquoise background encompasses strains having also telomeres with a ^5′^GGA^3′^ loop and a *gtpB-gtpA* operon, but with an additional type II *tap-tpg* operon. The light red background represents strains having the ancestral *gtpB-gtpA* operon, but also an additional type I *tap-tpg* operon and telomeres typified by a ^5′^CTTG^3′^ loop. The red arrow indicates a potential telomere replacement with a plasmid at the root of this sub-cluster.

**Figure 2:**
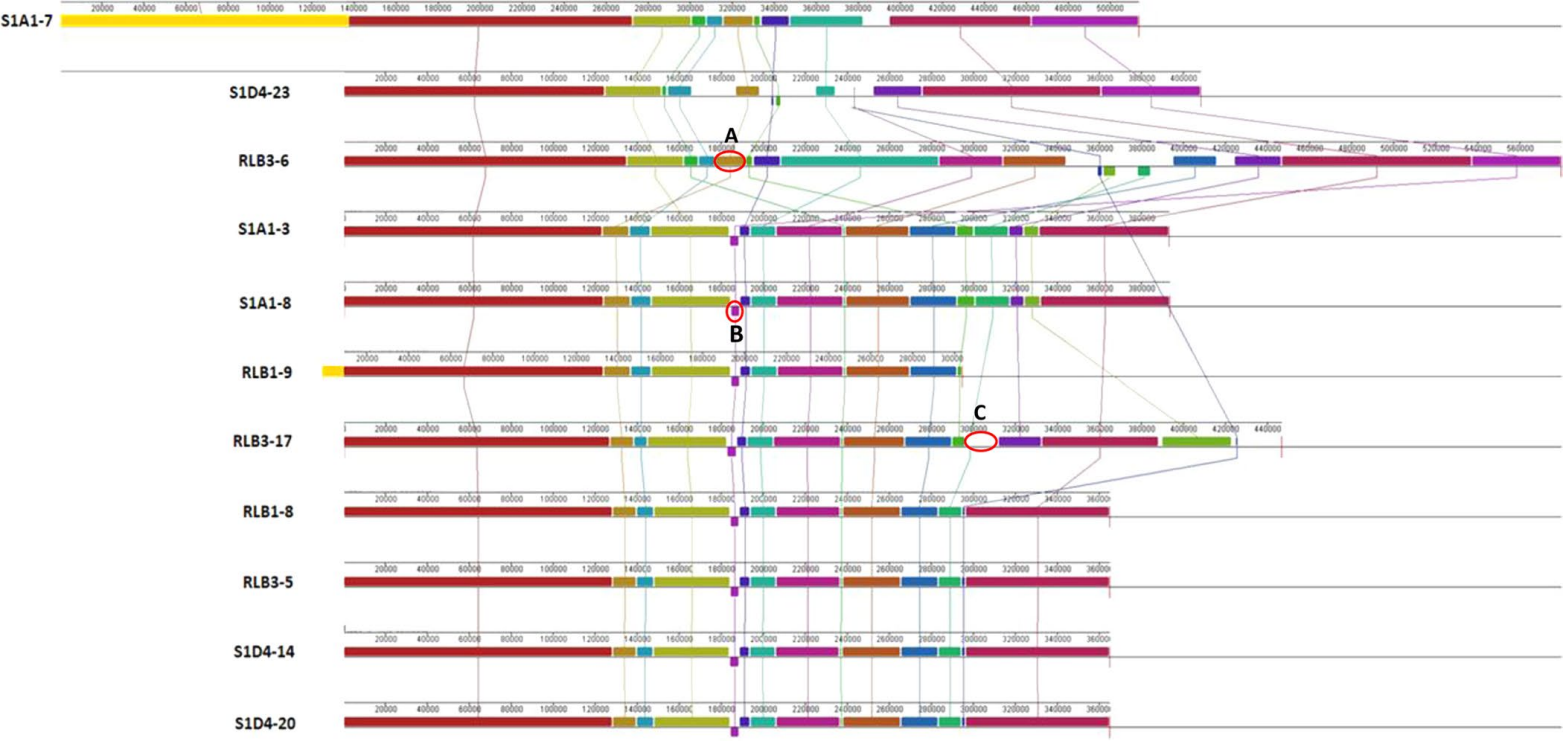
Identification of rearrangements events within the Terminal Inverted Repeats. The TIR sequences of the 11 strains were compared and visualized using the progressive Mauve algorithm for Windows. The alignment was performed on collinear sequences in which S1A1-7 TIR was used as reference sequence. Boxes with identical colors represent local collinear blocks (LCBs), indicating homologous DNA regions shared by two or more chromosomes without sequence rearrangements. Lines are drawn between LCBs in two adjacent species to indicate homologous regions. The placement of a block below the axis indicates an inversion event. Examples of rearrangement events occurring in the TIRs are highlighted by red circles; (**A**) Translocation event, (**B**) inversion event, (**C**) indel event.

This variability of the chromosomal TIR size revealed an intense plasticity of the terminal regions of the chromosome and this correlated with the different phylogenomic sub-clades (Fig. 1). Hence, while the size of TIRs is conserved between strains belonging to the sub-clade I (*e.g*., S1D4-20, RLB3-5, RLB1-8, and S1D4-14), it is highly variable within or between the other sub-clades (Table 1). For example, TIRs of strain RLB1-9 were shorter by 90 kb compared to those of its sister strains (*i.e*., S1A1-3 and S1A1-8) and lack the distal regions of the chromosome, but without the loss of linearity (not shown). Further recombination events (translocation, inversion and indels, labelled respectively A, B, C in Fig. 2) were revealed by global comparison of the TIRs of the 11 genomes using MAUVE (Fig. 2). Some regions are unique to one strain, suggesting that an has insertion occurred (*e.g*. Fig. 2, region C). Since this DNA region (16.7 kb) is not present elsewhere in the other 11 genomes, this suggested that this insertion was acquired through a horizontal gene transfer event.

**Figure 3:**
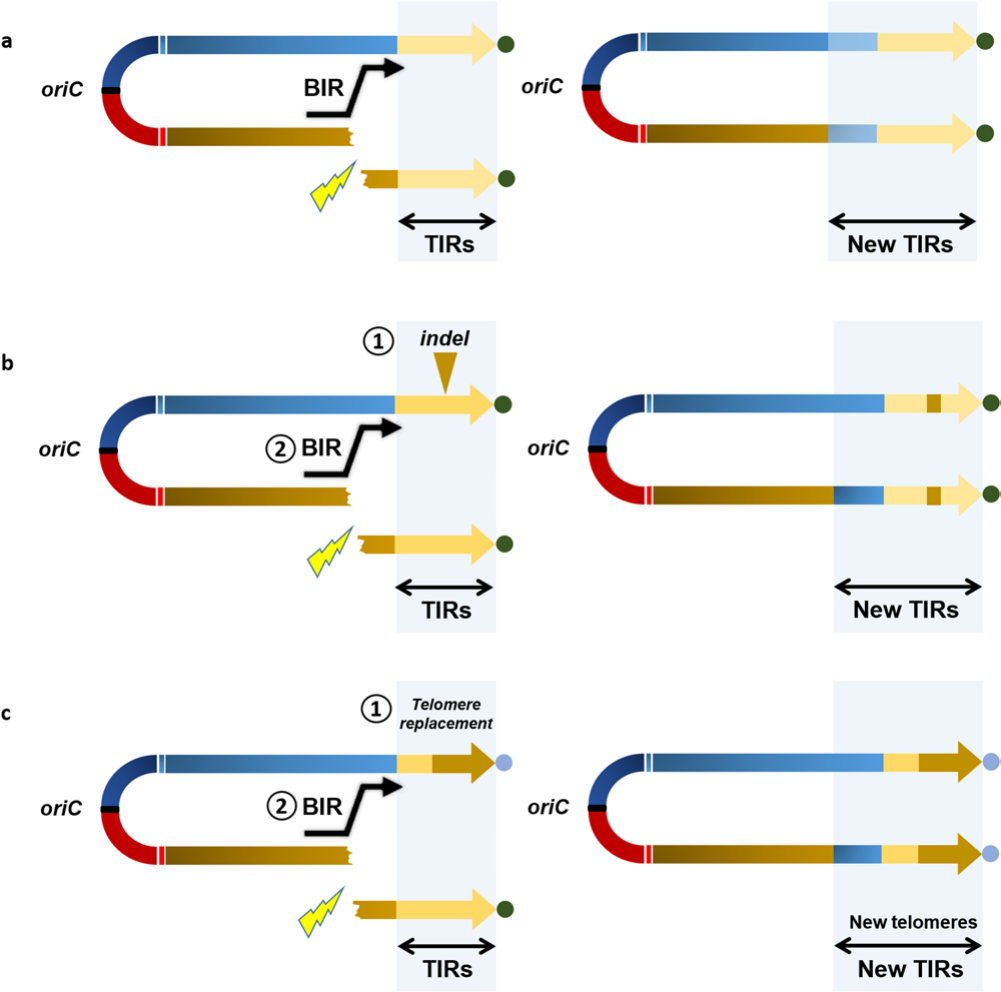
Inter-chromosomal arm recombination scenarios leading to homogenization of TIRs. Double-headed arrows represent *Streptomyces* linear replicons. The TIRs are highlighted with a light gray frame and the telomeres are represented by colored circles. The yellow flash symbols represent a DNA double strand break (DSB) requiring an upstream Break Induced Replication (BIR) event to rescue the broken replicon and to keep linearity. The BIR event uses the other arm as a matrix and recopy it until the end, including the telomere. If the DSB occurs within a TIR, the BIR will not change its size or sequence (not represented). (**a**) If the DSB occurs upstream the TIR, the BIR event will lead to the homogenization and extension in size of the TIRs. (**b**) An insertion/deletion event (indel) occurred (1) in one TIR (yellow triangle) before a DSB event. Like before, the BIR event (2) upstream the TIR will lead to a size increase, but will also propagate the indel event by homogenization. (**c**) In this case, a telomere replacement occurred in one arm (1) before the DSB. Following the BIR event (2), the arm homogenization will lead to a change of the telomere sequence.

Regardless of the nature of the recombination event, it generally modifies a single chromosomal end and consequently disrupts the TIRs. However, since we identify duplicated sequences, it is likely that a mechanism is in place to maintain two homogeneous copies of the identical TIR in a chromosome. Figure 3 depicts the potential recombination events required to maintain the chromosomal ends in a homogeneous state. This mechanism is reminiscent of the Break Induced Replication (BIR), which rescues broken chromosomes by recopying the intact arm through to the end (including the telomere), which is likely operating between the TIRs and maintaining identical TIR sequences. It also participates to shorten or increase the TIR size variability; hence, if the break point is located upstream or downstream the TIR border, then the size of the TIRs may increase or decrease respectively (Fig. 3a depicts a TIR increase). It was shown to be a powerful mechanism generating a high variability in TIR sizes under laboratory conditions for *S. ambofaciens*^10^. When an insertion occurs in a duplicated region (Fig. 3b), the same mechanism may lead to conversion of the original TIRs.

Given these results, the presence of TIRs at the ends of the *Streptomyces* chromosome appears to be a consequence of terminal recombinational activity. Reciprocally, their presence may also help rescue double-strand breaks occurring in the terminal part of the chromosome by providing an intact substrate for recombination repair^37^. Furthermore, terminal duplication may have functional consequences such as expression of specific gene function (*e.g*. specialized metabolite biosynthetic genes^38^; or may help in the maintenance a terminal cohesive structure such as the ‘racket-frame’ structure^39^.

### Identification of homologous recombination in the TIRs

**Figure 4:**
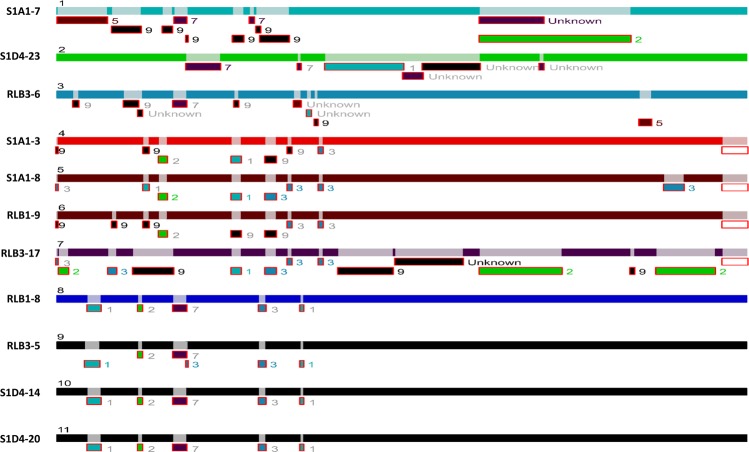
RDP4 analysis for DNA recombination and transfer within TIRs of the *Streptomyces* population Each bar represents the TIR sequence of one strain identified by its name and a number from 1 to 11. Regions of recombination events on each TIR are represented by a light shade of the corresponding TIR color. Sequence fragments from potential donors (parents from which recombinant sequence derived from) is depicted under each recombination region with the number corresponding to the donor strain. If the potential donor does not belong to the 11 strains, the fragment is assigned as unknown.

We inferred homologous recombination (HR) events by scanning the aligned sequences of the TIRs of the *Streptomyces* population with the RDP4 program. In total for the 11 genomes, 45 unique events were detected (Fig. 4). Strains of a same sub-clade mostly share the same HR events, where other strains exhibit specific HR events. Remarkably, RLB3-17, S1A1-7 and S1D4-23 account for 30 of the 45 unique HR events in these strains, providing evidence of the evolutionary history of the population. Common HR events within a sub-clade likely occurred in a recent common ancestor and spread vertically in these strains, where other strains may have accumulated increasing numbers of recombination events since the origin of the population. *Streptomyces* have already been shown to be recombinogenic, either at the genus^40^ or at the population level^41^. These previous studies were performed by calculating recombination frequencies with seven housekeeping genes (3,910 bp) located in the core genome. Here, due to the similarity of the strains HR events could be visualized between colinear TIRs (circa 408,938 bp). RDP suggests the most probable donor of a recombining DNA sequence, thus here the potential donor within our population. In one extreme case in these strains, the TIR of RLB3-17 seemed to have recombined several times with strains S1A1-7, S1D4-23, RLB3-6 and strains of sub-cluster I. This results in a mosaic structure confirming that the terminal regions are highly recombinogenic. It also highlights the massive gene flux previously described at the population level^23^ and that this population strains has experienced many gene transfer events.

### Telomere switching

Considering the high frequency of insertion and deletion events in the TIRs, we questioned the variability of the DNA extremities themselves, *i.e*., the telomere motifs, within our population. While insertions/deletions in TIRs require at least two recombination events to take place, the replacement of the most distal regions may take a single cross-over event. This terminal exchange results in the formation of a hybrid chromosome (*i.e*. with two different telomeres) further homogenized by inter-chromosomal arm recombination as depicted on Fig. 3c.

**Figure 5:**
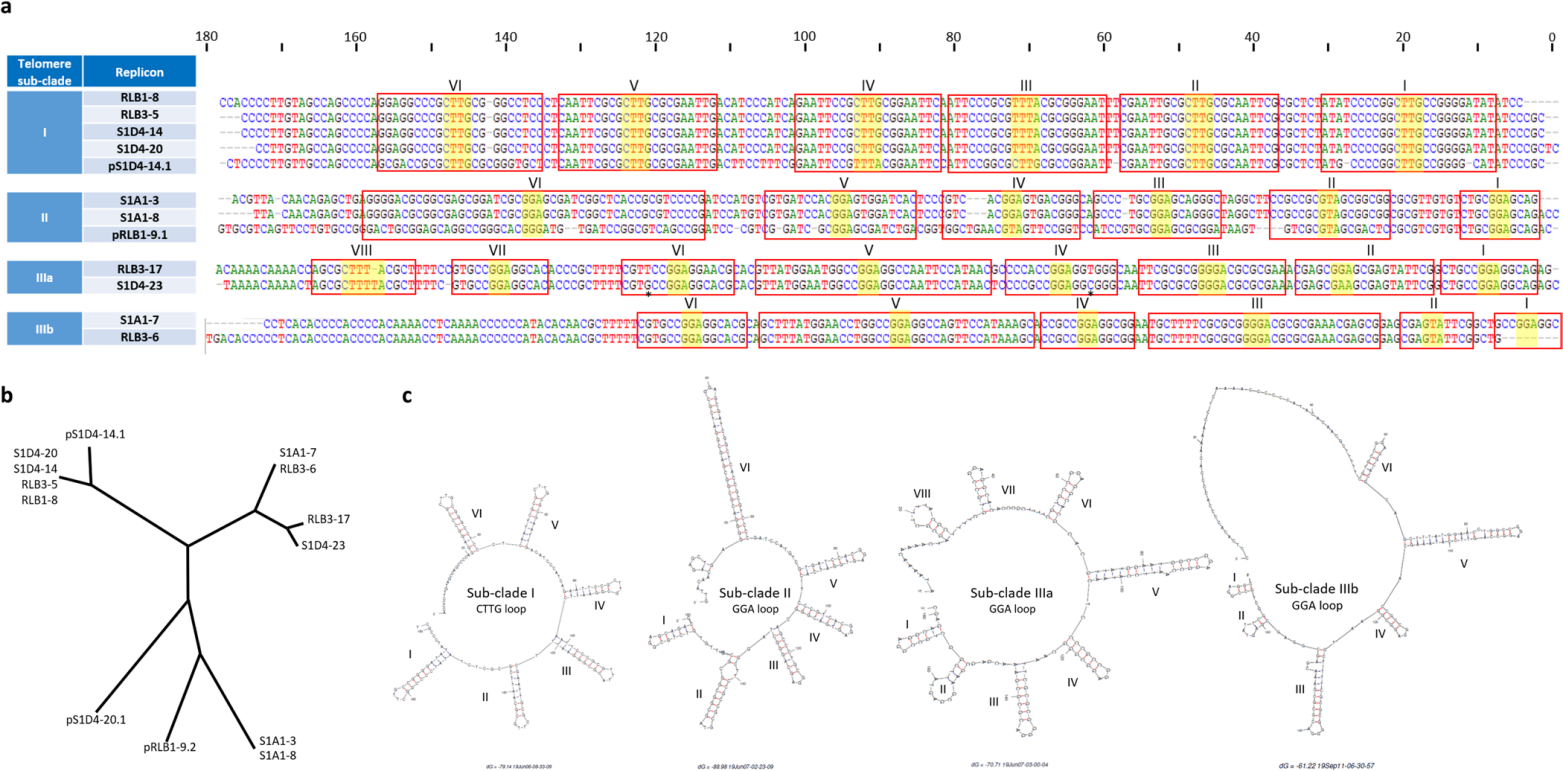
Comparison of the different chromosomal and plasmidic telomeres. (**a**) The terminal 180 nucleotide sequences from the 10 chromosomes and 2 linear plasmids were aligned. Four different groups designed in the table as telomere sub-clades were defined according to their sequence identities. Palindromic sequences are boxed and numbered in Arabic numerals above the sequences and the telomere loops are highlighted in yellow. Compensatory nucleotide changes within palindromes IV and VI of RLB3-7 telomere are highlighted in blue. The telomere sequence of plasmid pS1D4-20.1 could not be aligned with the others and is not presented. (**b**) Unrooted NJ phylogenetic tree built with the different telomere sequences. Positions with <80% site coverage in the alignment were eliminated enabling to have a total of 164 nucleotide positions in the final dataset. Bootstrap percentages are indicated on the branches. (**c**) The predicted secondary structures for a representative sequence of each telomere sub-cluster are represented. Two different loops (CTTG and GGA) can be observed at the top of the hairpin structures.

In our work, no specific sequencing approach was used to isolate and sequence the telomeres. However, in order to sequence the extremities of linear replicons, genomic DNA was initially prepared using a proteinase K step ensuring the degradation of terminal proteins bounded to DNA. To get as close as possible to the chromosomal end, we set out to walk on the chromosome towards the extremity by mining the sequencing reads (Illumina). This approach, enabled to extend from a few to several tens of nucleotides the previously published genomic sequences (see materials and methods) and *in silico* analyses (*mfold*) (Fig. 5c) of the 180 last nucleotides of each sequence revealed DNA hairpins and loops specific to *Streptomyces* telomeres. Despite we cannot rule out that the very last terminal nucleotides may still be missing in the final assemblies, however, this approach enabled to identify with confidence telomeric sequences for all chromosomes at the exception of RLB1-9. Regarding the other ten strains, four different telomere sequences were identified (Fig. 5a) with five to eight palindromic stems of variable length capped with conserved loop sequences (^5′^GGA^3′^ or ^5′^CTTG^3′^). Within the phylogenomic sub-clades I and II, respective strains shared identical telomere sequences (Fig. 5b) while their sequence identities declined to about 30% between sub-clades and were barely possible to align. In contrast, the telomeres of strains S1D4-23 and RLB3-6 forming the sub-clade III only shared weak identity (65%), where they were more closely related to the telomere sequences of RLB3-17 and S1A1-7 respectively that do not belong to sub-clade III (Fig. 5c), for example the S1D4-23 and RLB3-17 telomere sequences aligned almost perfectly (93% sequence identity) and exhibited only two mismatches that were compensatory mutations keeping the stem structures. Thus, telomere sequences defined two new telomere sub-divisions: IIIa with strains S1A1-7 and RLB3-6, and IIIb with strains RLB3-17 and S1D4-23 that were incongruent with the phylogenomic analysis (Fig. 5b). These data strongly support the hypothesis of telomere exchange within populations, and in this case that two of the strains (RLB3-6 and S1D4-23) acquired a new telomere, possibly from strains S1A1-7 or RLB3-17.

None of telomeres showed a significant nucleotide identity with the ‘archetypal’ telomeres (not shown). In contrast, telomeres of sub-clade I showed a strong identity (87%) with telomeres of linear plasmids including one of 92 kb from *Streptomyces dengpaensis* strain XZHG99 (GenBank accession number CP026653.1). The latter exhibits the end palindrome I (13 nt, ^5′^CCCGCTCCGCGGG^3′^) conserved in the archetypal telomere. Due to the limitations outlined above, we cannot rule out the presence of this palindrome at the ends of sub-clade I telomeres. Hence the last nucleotides of our sequences match the very first ones of the palindrome I sequence. However, since (i) there is no sequence homology with *S. coelicolor* and (ii) since the loop of the stems are capped with ^5′^CTTG^3′^ motifs instead of the classical ^5′^GCA^3′^ sheared pairing motif, we concluded that telomeres of sub-clade I constitute a new type of non-archetypal telomere. Further, the ends (over 50 nt) of the telomeres of sub-clade IIIa strains showed a strong homology with the atypical telomere of *S. griseus* 13350 and share with them the same sequence at the top of the stems (^5′^GGA^3′^). Finally, telomeres of sub-clade II showed 75% of nucleotide identity (over the last 3′ 150 nt of the telomere) with the ends of the *Streptomyces* sp. SirexAA-E chromosome, and possesses 6 stems capped with ^5′^GNA^3′^ loops (mostly ^5′^GGA^3′^).

In addition to the chromosomes, the telomere structures for the three linear plasmids (pRLB1-9.2, 106 kb, pS1D4-20.1, 394 kb; pS1D4-14.1, 112 kb) were identified. The telomere of pRLB1-9.2 possesses ^5′^GGA^3′^ loops (5 of 8 stem-loops, all sharing the classical G-A sheared pairing), the one of pS1D4-14.1 a ^5′^CTTG^3′^ loop at the top of five of the six last stem-loops and the one of pS1D4-20.1 is typified by an original ^5′^GCA^3′^ loop sequence (at the top of the last 3 of the 5 stem-loops). The novelty of this telomere was confirmed by that fact that no identity would be found with any sequence of the *nr* database.

The different telomere sequences in the population suggest that various recombination events occurred during the recent evolutionary history of the population. Hence, telomeres of strains of sub-clade I are typified by ^5′^CTTG^3′^ loops when other strains harbor ^5′^GNA^3′^ ones. Further, the telomere of pS1D4-14.1 (112 kb) are almost identical (97%) to that of the 92 kb-plasmid of *S. dengpaensis*. Given that pS1D4-14.1 telomeres also share a strong identity (84%) with sub-clade I chromosomes, it is tempting to hypothesize that a chromosome/plasmid replacement of the ancestral telomere loop ^5′^GGA^3′^ at the root of sub-clade I could explain the emergence of this telomere in the population (Fig. 1).

Although terminal recombination appears highly efficient to homogenise the terminal sequences and eliminate hybrid replicons, their presence has been reported previously. It has been shown that in *S. coelicolor* A3(2), both the chromosome (7.2 Mb) and a SCP1′ linear plasmid (1.85 Mb) are chimeric, generated by a single crossover between the wild-type chromosome and SCP1^42^. Similarly, in *S. cattleya* NRLL 8057, the linear chromosome and a megaplasmid appear to have exchanged telomeres leading to coexisting hybrid replicons^43^. Telomere plasticity seems to be common in *Borrelia* (spirochetes), the other main bacterial groups (38) possessing linear replicon^44^. This may result from telomere exchange as well as from telomere fusion, which may result from reversal of the telomere resolution reaction at the end of the replication process^45^. At the functional level, telomere recombination triggered by the formation of double strand breaks has also been associated to antigenic variation in *Trypanosoma brucei*^46^.

### Co-occurrence of telomere and terminal protein genes

Since terminal proteins (TP) interact in a specific manner with the telomere to achieve terminal replication^47^, the turnover of telomeres should be accompanied by that of the cognate terminal protein machineries. Therefore, we searched in the chromosomes and plasmids of our population for homologues of the archetypal Tap/Tpg genes described in *Streptomyces coelicolor* A3(2)^48^, of the atypical GtpB-GtpA of *Streptomyces griseus*^49^ as well as of the atypical Tac/Tpc terminal machinery of the linear plasmid SCP1 of *S. coelicolor* A3(2). No homologues of Tac/Tpc were identified (not shown), but we found that all the strains possessed a chromosomal homologue of the GtpB-GtpA encoding operon (c. 50% of amino acid identity with the *S. griseus* protein). Among the population, the conservation is high with amino acid identities higher than 98% for both gene products. This operon was likely inherited from the ancestor of the population (Fig. 1).

Using the archetypal Tap/Tpg of *S. coelicolor* as query sequences, we identified and distinguished two additional sets of genes including a Tpg homologue (called types I and II, Table 1) whose distribution followed the sub-clade phylogenies. Tpgs encoded in type I and type II sets showed amino acid identities of 48% and 59% with the archetypal Tpg, respectively. Type I and II Tpgs showed circa 40% of aa identity between them. All homologues exhibited the typical helix-turn-helix DNA binding domain associated to a nuclear localization signal (NLS) present in the archetypal Tpg although it was predicted at a slightly different location within the polypeptide in the type I Tpg product (Fig. S1). The type I Tpg also shared 78% of amino acid identity with the putative Tpg of *S. dengpaensis* (accession number AVH61776.1), that is much higher than with *S. coelicolor* Tpg and share the same NLS sequence and position. All the Tpgs proteins (type I and II) have almost the same size as the archetypal one (i.e. 175 aa).

In addition to the Tpgs, putative Tap proteins were also detected. In the type I gene set, a homologue of *S. coelicolor* A3(2) archetypal Tap was found with an amino acid identity of 51% (62% of similarity). A DNA binding domain was identified in the N-terminal domain of the Tap polypeptide in all homologues (not shown). Therefore, despite a common functional organization, the terminal complexes encoded by the archetypal and our type I gene set may recognize different telomeres.

In the type II gene set, beside the identified Tpg, we found a truncated version of a Tap gene (92 aa, C-ter, not shown) which appears to be a pseudogene. However, a long coding sequence immediately upstream encoded a polypeptide (648 aa) including an HTH motif in its N-terminal part just as in Tap proteins. Further, this polypeptide also contains a TPR/MLP domain (pfam07926) which is involved in the process of telomere length regulation in eukaryotes. This feature led us to hypothesise that this gene represents a candidate for the replacement of the original *tap* gene. We called it ‘Tap-alt’ (alt for alternative), and speculate that this atypical gene pair (Tpg/Tap-alt) may encode a terminal machinery able to handle atypical telomeres such as those found in sub-clade II.

Two of the three linear plasmids, pS1D4-14.1 and pS1D4-20.1, belonging to individuals of sub-clade I also harbour *tap-tpg* operons. While Tap and Tpg borne by pS1D4-14.1 strongly resembled those of the chromosomal genes of the same sub-cluster (i.e. 82% and 80%, respectively), pS1D4-20.1 encoded distantly related Tap-Tpg proteins (i.e. 38% and 46% respectively). In addition, these two Tap-Tpg pairs showed weak identities with archetypal proteins with 48% to 61% of identity. The presence of this atypical Tap-Tpg operon on pS1D4-20.1 plasmid is co-occurring with the unique telomere sequence in our population having ^5′^GCA^3′^ loops. It is tempting to suggest that this atypical terminal protein complex may take over the functioning of the unique telomere.

In contrast to linear plasmids of sub-clade I (pS1D4-14.1 and pS1D4-20.1), pRLB1-9.1 which belonged to strain RLB1-9 (sub-clade II) do not encode any Tap or Tpg homologue, and should benefit from host functions (type II Tap-alt/Tpg or GtpA-GtpB).

When the Tap/Tpg gene distribution is considered alongside telomere types, it is possible to hypothesise regarding the potential for co-evolution of telomeres and Tap/Tpg function within natural populations (Fig. 1). The presence of a type I Tap-Tpg locus is associated to the ^5′^CTTG^3′^ loop at the top of the stems of the telomere. This locus was identified in sub-clade I and on the plasmid pS1D4–14.1. Considering that the telomere sequences of chromosome and those of plasmid pS1D4–14.1 shared strong identities, it is tempting to speculate that a telomere replacement took place at the origin of this sub-clade and substituted the ancestral telomere (loop ^5′^GGA^3′^) by incoming the plasmid-borne one (^5′^CTTG^3′^). These non-archetypal and newly acquired telomeres likely require the presence of a specific terminal protein complex encoded by the atypical type I Tap-Tpg locus. Alternatively, these non-archetypal telomeres may be recognized by the *S. griseus* GtpAB like proteins as it would be in the remaining part of the population (that is present in all the strains and is the only TP complex in sub-clade III). Alternatively in sub-clade II, a new atypical complex encoded by the Tpg/Tap-alt cluster could be involved. The first hypothesis raises questions about the specificity of the interaction between the terminal protein complex and their cognate telomere. Since, the telomeres are rather different between sub-clades II, IIIa and IIIb, this would imply a high flexibility allowing wide recognition of telomeres. Alternatively, if the specificity of the telomere and of the terminal complex is tight, hence the Tpg/Tap-alt complex may be an alternative to handle the telomere, and this would strongly select for the simultaneous acquisition of a new telomere with its terminal complex. This could constitute a powerful selective force for organizing the genes encoding terminal complexes in the proximity of telomeres such that their simultaneous transfer ensures the functional characteristics of the telomere following transfer.

In conclusion, regardless of the terminal complexes supporting a range of telomeres types, the inconsistency between the phylogenomic and the telomere-based trees in the sub-clade III, suggests that terminal DNA exchanges have occurred (Fig. 1). Further, sub-clade I telomeres have undergone a probable replacement during diversification of the population through the exchange of telomeres with a linear plasmid. These events are the first report of a rapid turn-over of terminal region of the chromosomes in a natural population of *Streptomyces*.

## Supplementary Information

Supplementary Information.
