# Supplementary material for: Telomeric and sub-telomeric regions undergo rapid turnover within a Streptomyces population
Source: Sci Rep. 2020 May 7;10:7720. doi: 10.1038/s41598-020-63912-w (PMC7205883; doi:10.1038/s41598-020-63912-w)
Supplement: Supplementary file 1 — Supplementary Information. [file 41598_2020_63912_MOESM1_ESM.pptx]

## Slide 1
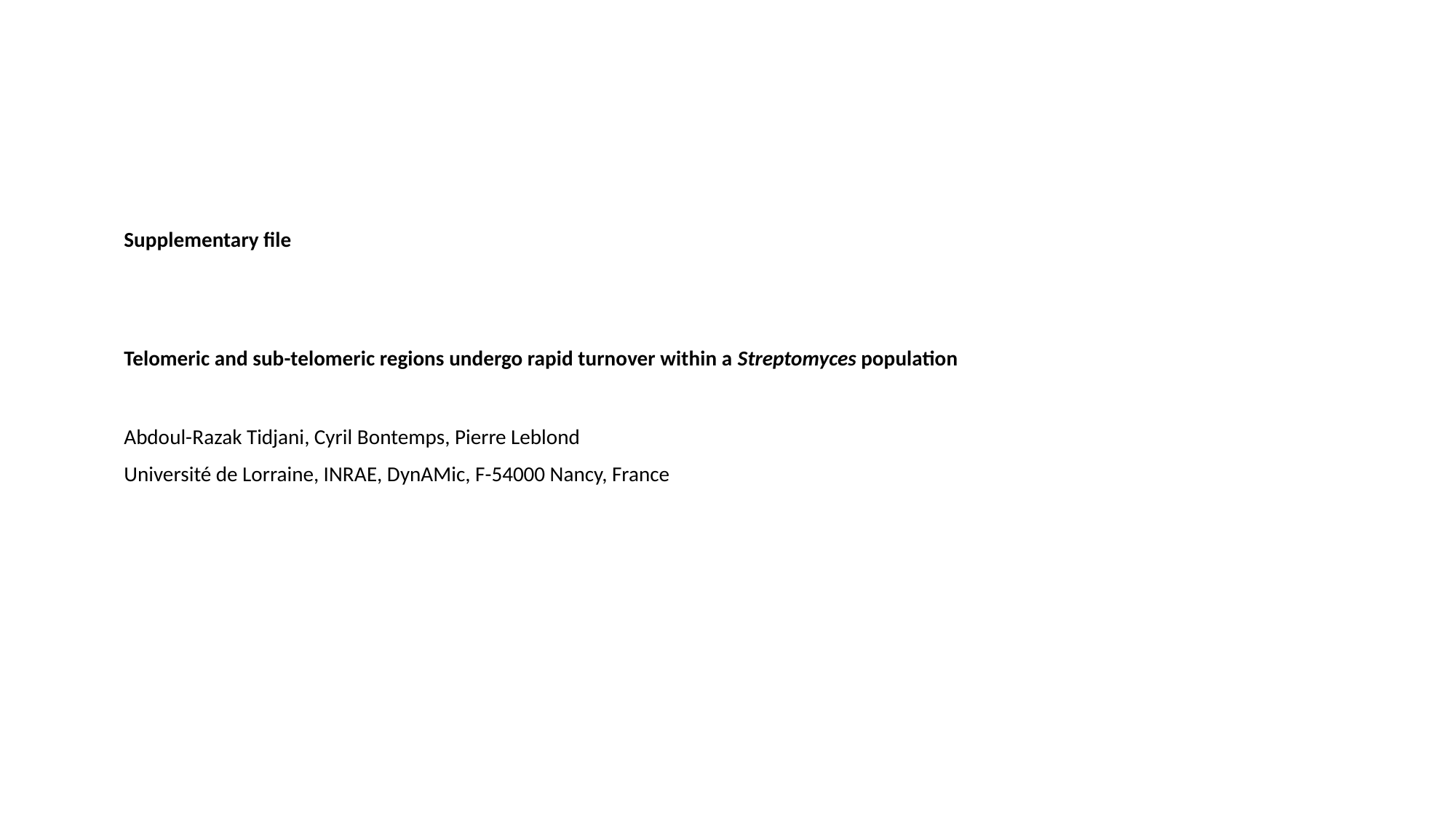

Supplementary file
Telomeric and sub-telomeric regions undergo rapid turnover within a Streptomyces population
Abdoul-Razak Tidjani, Cyril Bontemps, Pierre Leblond
Université de Lorraine, INRAE, DynAMic, F-54000 Nancy, France

## Slide 2
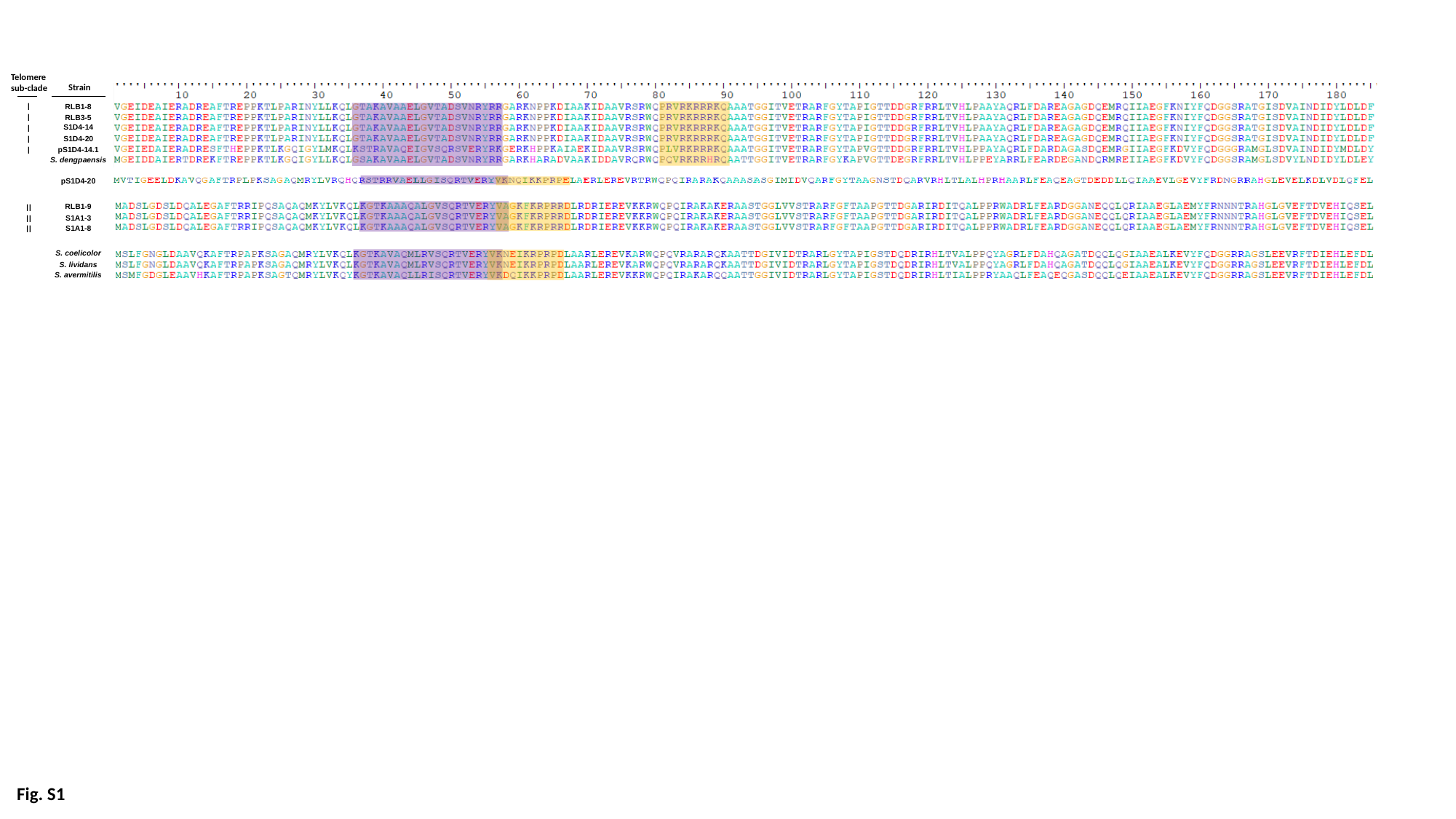

Telomere
sub-clade
I
I
I
I
I
II
II
II
Strain
RLB1-8
RLB3-5
S1D4-14
S1D4-20
pS1D4-14.1
S. dengpaensis
pS1D4-20
RLB1-9
S1A1-3
S1A1-8
S. coelicolor
S. lividans
S. avermitilis
Fig. S1
